# Characterization of Rab32- and Rab38-positive lysosome-related organelles in osteoclasts and macrophages

**DOI:** 10.1016/j.jbc.2023.105191

**Published:** 2023-08-23

**Authors:** Kazuya Noda, Shiou-Ling Lu, Siyu Chen, Kanako Tokuda, Yangjie Li, Feike Hao, Yoh Wada, Ge-Hong Sun-Wada, Shinya Murakami, Mitsunori Fukuda, Takashi Itoh, Takeshi Noda

**Affiliations:** 1 Department of Oral Cell Biology, Center for Frontier Oral Science, Graduate School of Dentistry, Osaka University, Osaka, Japan; 2 Department of Periodontology, Graduate School of Dentistry, Osaka University, Osaka, Japan; 3 Graduate School of Frontier Biosciences, Osaka University, Osaka, Japan; 4 Department of Biological Sciences, Institute of Scientific and Industrial Research, Osaka University, Osaka, Japan; 5 Center for Infectious Disease Education and Research, Osaka University, Osaka, Japan; 6 Department of Biochemistry, Faculty of Pharmaceutical Sciences, Doshisha Women's College, Kyoto, Japan; 7 Department of Integrative Life Sciences, Graduate School of Life Sciences, Tohoku University, Miyagi, Japan

**Keywords:** Rab32, Rab38, osteoclast, macrophage, lysosome-related organelles

## Abstract

Both the biogenesis and functions of osteoclasts and macrophages involves dynamic membrane traffic. We screened transcript levels for Rab family small GTPases related to osteoclasts and identified Rab38. Rab38 expression is upregulated during osteoclast differentiation and maturation. In osteoclasts, both Rab38 and its paralog, Rab32, colocalize to lysosome-related organelles (LROs). In macrophages, Rab32 is also found in LROs. LROs are part of the endocytic pathway but are distinct from lysosomes. After receptor activator of NF-κB ligand stimulation, LROs contain cathepsin K and tartrate-resistant acid phosphatase inside and help both proteins to accumulate around bone resorption pits. After osteoclast maturation, these enzymes are hardly found within LROs. In macrophages derived from Rab32 and Rab38 double knockout mice, both acidification and V-ATPase *a*3 localization were severely compromised. Both the double knockout macrophage and bafilomycin-treated wildtype macrophage show an increase in Lamp1-positive organelles, implying that biogenesis of lysosomes and LROs are related. These results indicate that Rab32 and Rab38 both play a crucial role in LRO biogenesis in macrophages and in osteoclasts.

Human bones are maintained through constant remodeling, and an imbalance in bone homeostasis causes various diseases, including osteopetrosis, osteoporosis, and periodontal disease (1, 2). To understand the etiology of these diseases, we must deepen our understanding of osteoclast function. Osteoclasts are syncytia, giant multinucleated cells generated from the fusion of macrophages (3). Two cytokines, macrophage colony stimulating factor and receptor activator of NF-κB ligand (RANKL), play important roles in macrophage differentiation (3). Mature osteoclasts form an actin ring structure, called the sealing zone, that binds tightly to the bone surface (4, 5). Osteoclasts secrete a variety of proteases and acids into the ruffled border within the sealing zone, which form a bone resorption pit called Howship’s lacuna, to degrade collagen and the bone matrix (6). The dynamic movement of organelles and protease-containing vesicles are involved in resorption processes (7, 8). Additionally, in macrophages, materials taken up by phagocytosis are transported to lysosomes *via* the endocytic pathway. Furthermore, both in osteoclasts and macrophages, specialized organelles called lysosome-related organelles (LROs) are involved in this membrane traffic and have features similar to those of both endosomes and lysosomes, but also have cell type-specific functions (9).

Rab family proteins are members of the Ras family of low molecular weight G proteins that function as molecular switches by cycling between active GTP-bound and inactive GDP-bound forms (10, 11). In the active form, Rab proteins bind to effector proteins and execute various functions such as membrane trafficking, organelle biosynthesis, and cell adhesion (12).

There are at least 60 types of Rab proteins in humans, and there are several reports concerning the involvement of Rab proteins in osteoclast biogenesis. For example, Rab7, which regulates lysosome trafficking, is involved in ruffled border formation and bone resorption (13). Lissencephaly-1, which binds to cytoplasmic dynein and regulates its transport, interacts with Rab7 and Pleckstrin homology domain-containing family M member 1 (14). Rab3D regulates secretory vesicle trafficking, and Rab3D KO mice show both abnormal ruffled border formation and increased bone mass (15). Rab3D interacts with T-complex testis-expressed-1, a subunit of the light chain of cytoplasmic dynein that controls secretory vesicle trafficking (16). Rab13 has also been proposed to be involved in secretory vesicle trafficking (17). Furthermore, Rab27A, which is involved in transporting LROs and secretory vesicles, is also involved in osteoclast differentiation and resorption (18). By contrast, Rab11a, b, and Rab44 all function negatively in osteoclast differentiation (19, 20, 21).

In this study, we aimed to determine the characteristics of macrophages and osteoclasts from the viewpoint of membrane trafficking by focusing on Rab proteins. We found that Rab32 and Rab38 both play a crucial role in these cells.

## Results

### Screening of Rab proteins associated with macrophages and osteoclasts

**Figure 1:**
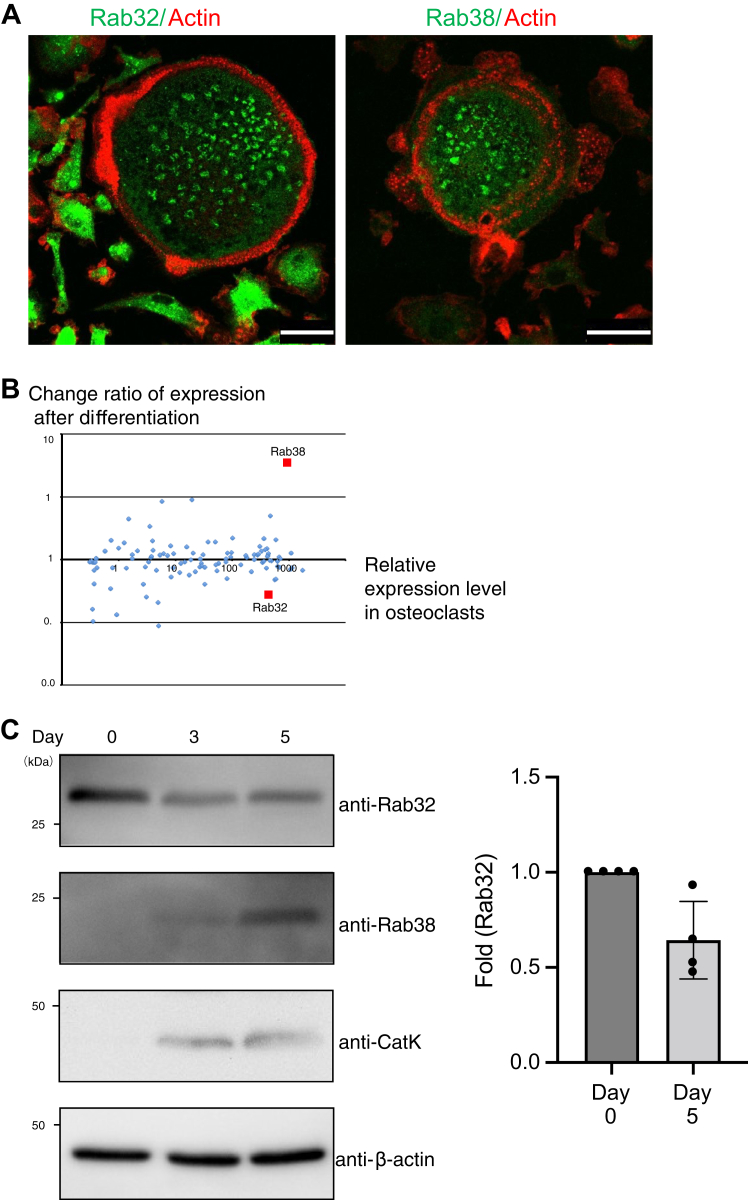
**Rab32 and Rab38 expression patterns in macrophages and osteoclasts**. *A*, macrophages cultured on cover glasses were induced to differentiate into osteoclasts by RANKL treatment. Cells were fixed and stained with endogenous Rab32 (Santa Cruz, B-4, sc-3901784) or Rab38 (Santa Cruz, A-8, sc-3901768) and actin. The localization of Rab32-or Rab38-positive ring structures within the actin ring was determined by confocal fluorescence microscopy. Scale bar: 25 μm. *B*, gene expression was determined by microarray using mRNA derived from macrophages and cells on day 6 of RANKL stimulation. Expression levels and changes in expression rates (arbitrary units) of each Rab mRNA in osteoclasts are shown. *C*, macrophages (RANKL-stimulation day 0, day 3, and day 5) were collected. Cell lysates were subjected to Western blotting with specific antibodies to detect the expression of Rab32, Rab38, cathepsin K, and actin protein. The band intensity of the Rab32 signal was measured using the ImageJ software. The average and SD of four independent experiments are shown. RANKL, receptor activator of NF-κB ligand.

To screen Rab proteins that function in both macrophages and osteoclasts, we stably expressed 55 Rab proteins N-terminally tagged with GFP in bone marrow–derived macrophages collected from 8-week-old male mice. The macrophages were differentiated into osteoclasts by RANKL treatment and observed by confocal microscopy. Some of the Rab proteins showed a ring-like localization pattern in both macrophages and osteoclasts (Table 1). Signals that did not exhibit any morphological character other than puncta in confocal microscopy are described as dot-like structures. Vacuole-like signals with attenuated interior area signal are described as ring-like structures. Rab proteins that showed a ring-like pattern were selected and cultured on dentin plates to induce resorption pit formation. Hence, part of GFP-Rab7, Rab9A, Rab13, Rab32, Rab35, and Rab38 signals accumulated inside the actin ring that surrounds absorption pit, and those were considered potential candidates for linkage to osteoclast functioning (Fig. S1*A* and data not shown). Among them, Rab32- and Rab38-positive ring-like signals stained with immunofluorescence were located at a rate of 37% (11/30) and 56% (19/34) of osteoclast actin rings, respectively (Fig. 1*A*).

**Table 1:** The localization pattern of each Rab protein

Rab isoform	Subcellular localization	Rab isoform	Subcellular localization
Rab1A	Perinuclear+dot/ring	Rab18	Perinuclear+cytoplasm
Rab1B	Perinuclear+dot/ring	Rab19	Perinuclear+dot/ring
Rab2A	Perinuclear	Rab20	Perinuclear+dot/ring
Rab2B	Perinuclear	Rab21	Perinuclear+dot/ring
Rab3A	Perinuclear	Rab22A	Perinuclear+dot/ring
Rab3B	Perinuclear+dot/ring	Rab22B	Perinuclear+dot/ring
Rab3C	Perinuclear+dot/ring	Rab23	Dot/ring+plasma membrane
Rab3D	Perinuclear+dot/ring	Rab24	Perinuclear+cytoplasm
Rab4A	Dot/ring	Rab25	Dot/ring
Rab4B	Dot/ring	Rab26	Dot/ring
Rab5A	Dot/ring	Rab27A	Not detected
Rab5B	Dot/ring	Rab27B	Perinuclear+dot/ring
Rab5C	Dot/ring	Rab28	Perinuclear
Rab6A	Perinuclear+plasma membrane	Rab29	Perinuclear
Rab6B	Perinuclear+plasma membrane	Rab30	Perinuclear
Rab6C	Not detected	Rab32	Perinuclear+dot/ring
Rab7	Dot/ring	Rab33A	Perinuclear+dot/ring
Rab8A	Perinuclear+plasma membrane	Rab33B	Perinuclear
Rab8B	Perinuclear+plasma membrane	Rab34	Perinuclear+plasma membrane
Rab9A	Dot/ring	Rab35	Dot/ring+plasma membrane
Rab9B	Perinuclear+dot/ring	Rab36	Perinuclear
Rab10	Perinuclear+dot/ring	Rab37	Dot/ring
Rab11A	Dot/ring+plasma membrane	Rab38	Perinuclear+dot/ring
Rab11B	Dot/ring+plasma membrane	Rab39A	Dot/ring
Rab12	Perinuclear+dot/ring	Rab39B	Dot/ring
Rab13	Perinuclear+plasma membrane	Rab40A	Not detected
Rab14	Dot/ring	Rab40B	Perinuclear+nuclear
Rab15	Perinuclear	Rab40C	Not detected
Rab17	Perinuclear+dot/ring+plasma membrane	Rab41	Perinuclear
		Rab42	Dot/ring

The expression was specific for each of these proteins. Dot-like structures refer to signals that did not exhibit any morphological character other than puncta in confocal microscopy. Ring-like structures refer to vacuole-like signals with a signal-less area inside it.

In a different line of screening, we sought Rab proteins expressed in osteoclasts. Macrophages were induced to differentiate into osteoclasts with RANKL. We used a microarray to determine mRNA expression levels before and after the sixth day of RANKL stimulation. The expression of osteoclast-associated genes, tartrate-resistant acid phosphatase (TRAP) (22), cathepsin K (CatK) (23), dendritic cell-specific transmembrane protein (24), and calcitonin receptor (25) were upregulated during osteoclast differentiation as previously reported (Fig. S1*B*). As for Rab protein genes, Rab38 levels were about 40 times higher in osteoclasts than in macrophages (Fig. 1*B*). Consistently, Rab38 expression is reportedly regulated by NFATc, the master transcriptional factor during osteoclast differentiation (26). Rab32, a Rab38 paralog, is also highly expressed in osteoclasts, although its expression level decreased about 70% when osteoclasts undergo differentiation (Fig. 1*B*) (27, 28, 29). We found by quantitative PCR that Rab38 expression levels increased by 48 times while Rab32 expression levels dropped by about half (Fig. S1*C*).

We also determined the expression of endogenous Rab32 and Rab38 protein before and after RANKL stimulation by Western blotting using specific antibodies (Fig. 1*C*). Rab32 was expressed with or without RANKL stimulation but at slightly lower levels following stimulation, just like mRNA (Fig. 1*C*). While Rab38 could not be detected before RANKL stimulation, its expression increased slightly on day 3 when cathepsin K was already fully upregulated. On day 5, Rab38 signal increased to a greater extent. Based on these results, we chose to focus on Rab32 and Rab38 (hereafter referred to as Rab32/38) in macrophages and osteoclasts.

### Rab32 and Rab38 colocalize in osteoclasts

**Figure 2:**
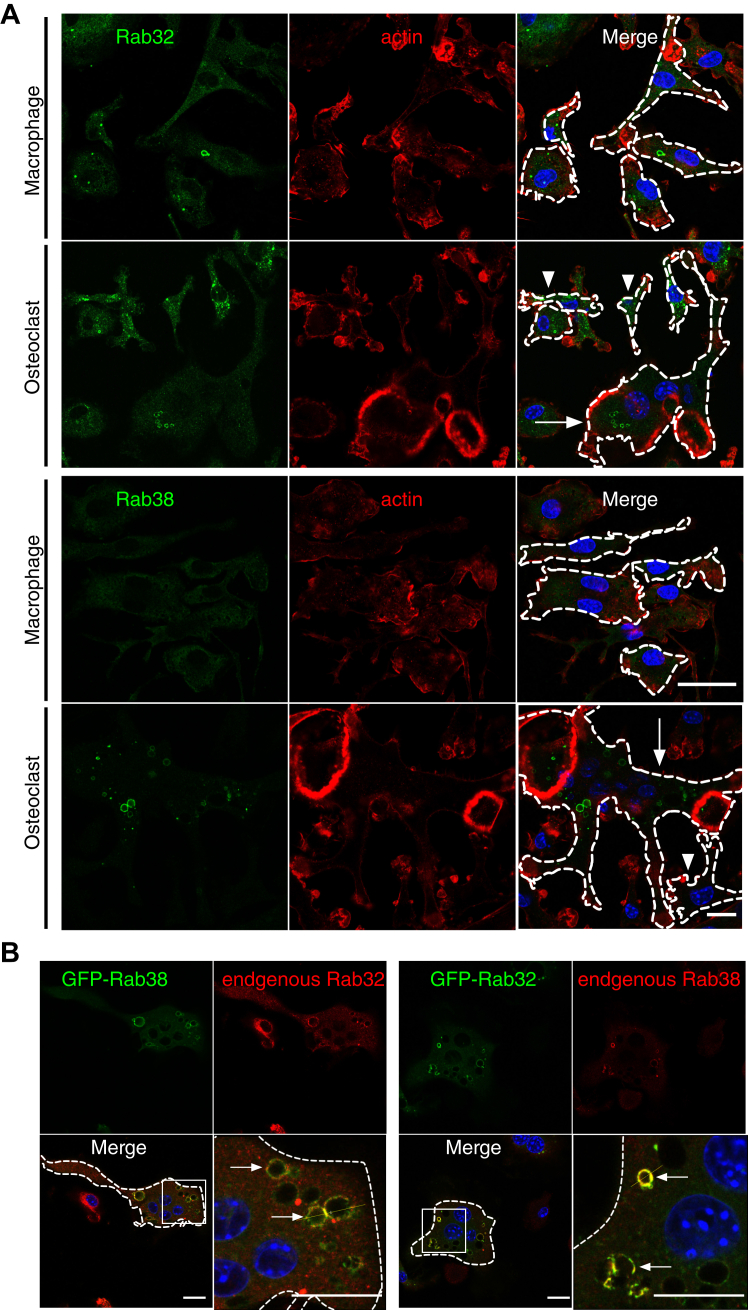
**Rab32 and Rab38 colocalize in osteoclasts**. *A*, cells (macrophages) on day 0 and day 5 of RANKL stimulation were labeled with Rab32 and Rab38 antibodies and observed by confocal fluorescence microscopy. *Arrows* indicate multinucleated osteoclasts, and *arrowheads* indicate mononucleated osteoclasts. *B*, macrophages were transduced with lentivirus for either GFP-Rab32 or GFP-Rab38 expression and induced to differentiate into osteoclasts by RANKL stimulation. After fixation, cells were labeled with antibodies against either Rab38 or Rab32 and observed by confocal fluorescence microscopy. *Arrows* indicate colocalization. Scale bar: 25 μm. RANKL, receptor activator of NF-κB ligand.

Next, we studied the intracellular localization of Rab32/38 in greater detail. Immunofluorescence labeling of endogenous Rab32 showed a dot- and ring-like structure in macrophages (Fig. 2*A*). We observed dot- and ring-like structures in multinuclear osteoclasts as well as localization inside absorption pits as described above (Fig. 2*A*). Endogenous Rab38 was not observed in macrophages, consistent with its apparent lack of expression in macrophages, while dot- and ring-like structures were seen in multinucleated osteoclasts (Fig. 2*A*). Likewise, in osteoclasts expressing either GFP-Rab32 or GFP-Rab38 and cultured on dentin, both GFP-fusion proteins showed mainly dot- and ring-like localization patterns (Fig. S2). GFP-Rab32 and endogenous Rab38 mostly colocalized in multinucleated osteoclasts (Figs. 2*B* and S3*A*). Similarly, GFP-Rab38 and endogenous Rab32 were mostly colocalized in osteoclasts (Figs. 2*B* and S3*B*). These results indicate that Rab32 and Rab38 localize to the same organelle in osteoclasts.

### Rab32-positive organelles show differing properties from lysosomes in the endocytic pathway

**Figure 3:**
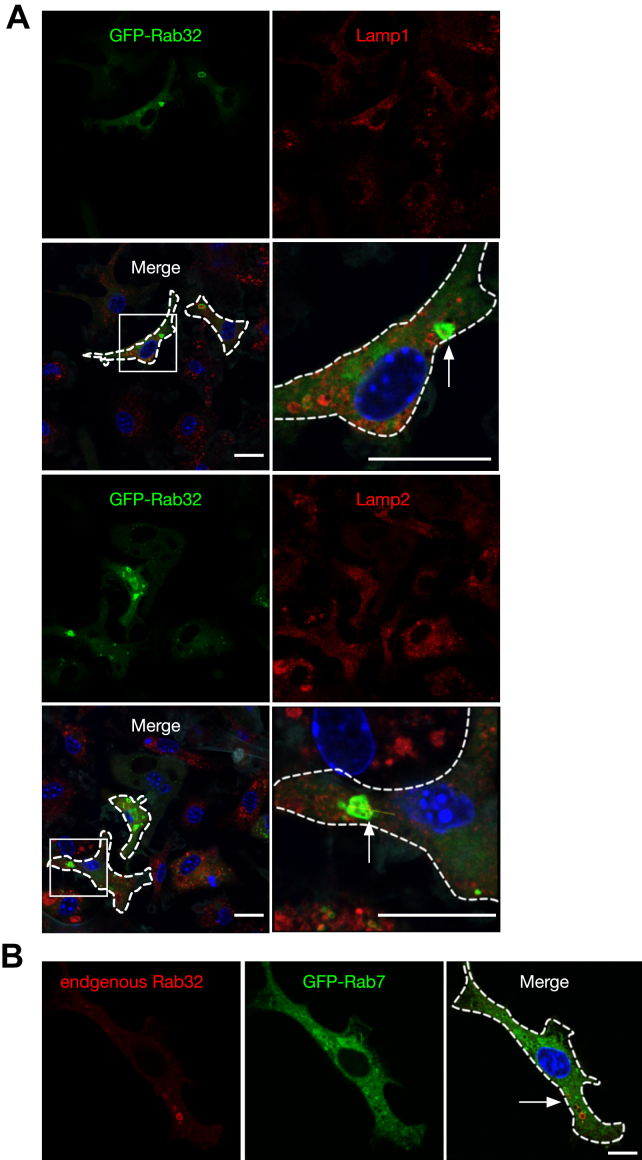
**Rab32-positive organelles are positive for Lamp1 and Lamp2 but deficient in Rab7 in macrophages.***A*, macrophages were transduced with lentivirus for either GFP-Rab32 or GFP-Rab38 expression. Transduced cells were fixed and labeled with antibodies against a lysosomal marker, either Lamp1 or Lamp2, and observed by confocal fluorescence microscopy. *Arrows* indicate colocalization. *B*, GFP-Rab7 expressing macrophages were labeled with anti-Rab32 antibody and observed by confocal fluorescence microscopy. *Arrows* indicate colocalization. Scale bar: 25 μm. Lamp1, lysosomal associated membrane protein 1; RANKL, receptor activator of NF-κB ligand.

To determine the relationship between Rab32- and Rab38-positive organelles and early endosomes, we determined if either GFP-Rab32 or GFP-Rab38 colocalize with EEA1, an early endosome marker, in macrophages and osteoclasts. Both GFP-Rab32 and GFP-Rab38 did not colocalize with EEA1, excluding the possibility of localization to early endosomes (Fig. S4). When we observed the localization of GFP-Rab32 and lysosomal marker proteins Lamp1 (lysosomal-associated membrane protein 1) and Lamp2 in macrophages, most GFP-Rab32-positive organelles were also positive for Lamp1 and Lamp2 (Figs. 3*A* and S3, *C* and *D*). When we compared the localization of GFP-Rab7, a lysosomal and late endosomal marker, with that of Rab32, however, we found that the two proteins showed little evidence of colocalization. Instead, most Rab32 showed a distinct dot- and ring-like localization (Figs. 3*B* and S3*E*).

**Figure 4:**
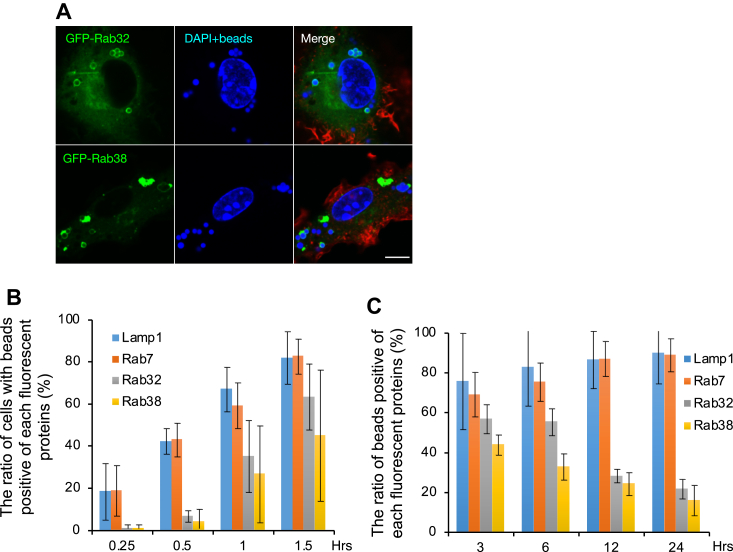
**Phagocytosed fluorescent beads are inside Rab32/38-positive organelles.***A*, fluorescent beads were added to the macrophage culture medium expressing each fluorescent protein, fixed after 90 min, and observed by confocal fluorescence microscopy. The scale bar: 10 μm. *B*, in macrophages expressing GFP-Rab7, -Rab32, -Rab38, or Lamp1-RFP, fluorescent beads were added to the medium and fixed at each time point, and the cells were observed by confocal fluorescence microscopy. The percentage of cells with fluorescent beads within each marker-positive organelle was counted for at least 30 cells at each time point in a blinded manner. *C*, in macrophages expressing GFP-Rab7, -Rab32, -Rab38, or Lamp1-RFP, fluorescent beads were added to the medium, fixed at each time point, and cells were observed by confocal fluorescence microscopy. The percentage of fluorescent beads localized inside the organelle was counted for at least 10 cells per time point in a blinded manner. In figures *B* and *C*, the graphs show the SD of four independent experiments. Lamp1, lysosomal associated membrane protein 1.

Next, we investigated the relationship between Rab32-positive organelles and the endocytic pathway. Fluorescent beads added to the medium were phagocytosed by macrophages and observed by confocal fluorescence microscopy. As expected, the fluorescent beads were observed within Rab5-positive early endosomes, Rab7-positive late endosomes, lysosomes, and Lamp1-positive lysosomes, which make up the endocytic pathway (data not shown). In addition, fluorescent beads were seen inside GFP-Rab32-positive organelles (Fig. 4*A*). Similarly, when fluorescent dye-labeled dextran was added to observe liquid phase endocytosis, we observed dextran localization inside GFP-Rab32-positive organelles (data not shown). These results indicate that Rab32-positive organelles are part of the endocytic pathway in macrophages.

Next, we measured the rate at which fluorescent beads became localized within each organelle marker-positive organelle. The percentage of cells with fluorescent beads inside GFP-Rab32 positive structure increased up to 3 h after they were introduced, slightly slower than GFP-Rab7 and Lamp1-RFP (Fig. 4*B*). When the incubation time was further extended, almost all the incorporated fluorescent beads became positive for GFP-Rab7 and Lamp1-RFP, indicating lysosomal localization (Fig. 4*C*). However, the percentage of GFP-Rab32-positive fluorescent beads decreased after 3 h and finally reached less than 20% (Fig. 4*C*). Although Rab38 is not expressed in macrophages, GFP-Rab38 showed similar results to GFP-Rab32 (Fig. 4, *B* and *C*). These results suggest that Rab32-positive organelles in macrophages are just a part of the Lamp1-positive organelle in the endocytic pathway and are distinct from authentic lysosomes.

### Rab32- and Rab38-positive organelles exhibit different properties from lysosomes during osteoclast maturation

**Figure 5:**
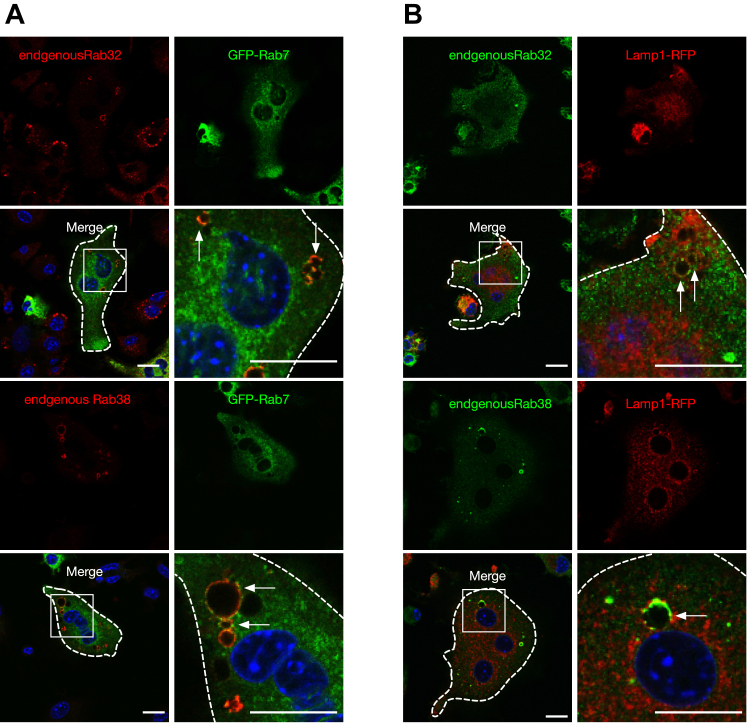
**Endogenous Rab32/38 partially colocalizes with GFP-Rab7 in osteoclasts**. *A*, macrophages were transduced with lentivirus for GFP-Rab7 expression and induced to differentiate into osteoclasts by RANKL stimulation. After fixation, the cells were labeled with Rab32 and Rab38 antibodies and observed by confocal fluorescence microscopy. *Arrows* indicate colocalization. *B*, macrophages were transduced with lentivirus for Lamp1-RFP expression and induced to differentiate into osteoclasts by RANKL stimulation. After fixation, cells were labeled with Rab32 and Rab38 antibodies and observed by confocal fluorescence microscopy. *Arrows* indicate colocalization. Scale bar: 25 μm. Lamp1, lysosomal associated membrane protein 1; RANKL, receptor activator of NF-κB ligand.

Next, to determine the relationship between Rab32- and Rab38-positive organelles and lysosomes in osteoclasts, we observed the localization of GFP-Rab7 transgenic osteoclasts and endogenous Rab32 and Rab38 (Fig. 5*A*). We found that the Rab32/38-positive ring-like structures are mostly associated with GFP-Rab7 signals; however, the signals were punctuated but not distributed in ring-like patterns, which suggests that they are associated with a subdomain of the organelle. When we transduced macrophages simultaneously with GFP-Rab32 and Rab38 and Strawberry-Rab7 expression and induced them to differentiate into osteoclasts, most of the resulting transductants showed similar colocalization (Fig. S5). Furthermore, we observed that Lamp1-RFP usually colocalized with endogenous Rab32 and Rab38 (Fig. 5*B*).

**Figure 6:**
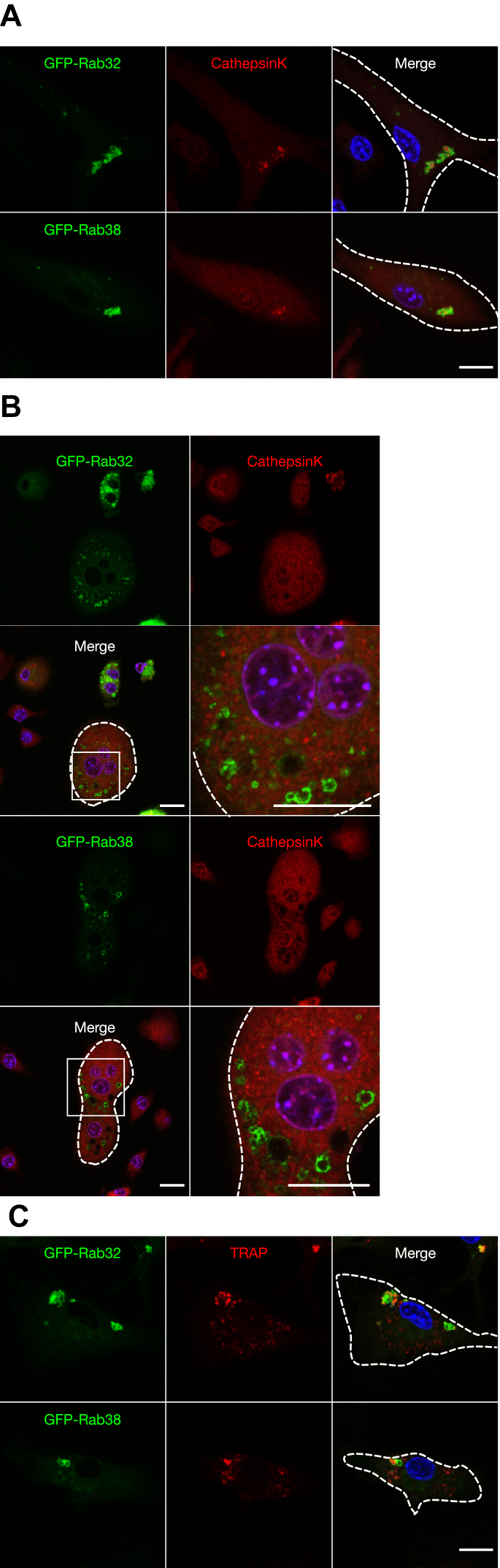
**Localization of cathepsin K inside Rab32- and Rab38-positive organelles after osteoclast induction***. A* and *B*, macrophages were genetically transfected with GFP-Rab32/38 and induced to differentiate into osteoclasts by RANKL stimulation. Magic Red Cathepsin K was added 30 min before fixation and observed by confocal fluorescence microscopy. *C*, macrophages were transduced with either GFP-Rab32 or GFP-Rab38 expression and RANKL stimulation was performed. After fixation, the cells were labeled with TRAP antibody and observed by confocal fluorescence microscopy. Scale bar: 25 μm. RANKL, receptor activator of NF-κB ligand; TRAP, tartrate-resistant acid phosphatase.

Additionally, we determined the localization of cathepsin K, a protease found within lysosomes that is released into resorption pits during bone resorption (23). The cells were treated with Magic Red CatK, which labels sites where cathepsin K is active. In mononuclear cells after RANKL stimulation, most of the signals were localized inside GFP-Rab32- and GFP-Rab38-positive organelles (Fig. 6*A*). These signals were barely observed in cells before RANKL treatment (data not shown). In multinucleated osteoclasts, however, the signals were scarcely observed inside GFP-Rab32- and GFP-Rab38-positive organelles (Fig. 6*B*). We also determined cathepsin K localization by immunofluorescence using an anti-cathepsin K antibody. Again, cathepsin K was not observed inside GFP-Rab32- and GFP-Rab38-positive organelles in multinucleated osteoclasts (Fig. S6*A*). When Magic Red CatB, which indicates cathepsin B activity, was added, most of the signal was observed within the GFP-Rab32- and GFP-Rab38-positive organelles in mononuclear cells similar to the localization of Magic Red CatK (Fig. S6*B*). By contrast, multinucleated osteoclasts did not show the cathepsin B signal within most GFP-Rab32- and GFP-Rab38-positive organelles (Fig. S6*C*). Furthermore, TRAP, an osteoclast-specific marker enzyme, was observed inside the GFP-Rab32/38-positive organelles in most mononuclear cells after RANKL stimulation (Fig. 6*C*). These results strongly suggest that during osteoclast maturation, Rab32- and Rab38-positive organelles in mononuclear cells contain cathepsin K, cathepsin B, and TRAP, but that the protease is lost after osteoclast maturation, and most may exist apart from lysosomes.

In summary, even after osteoclasts mature, Rab32- and Rab38-positive organelles eventually exhibit properties distinct from lysosomes.

### Rab32 and Rab38 are involved in acidification of the lysosome by affecting V-ATPase localization

**Figure 7:**
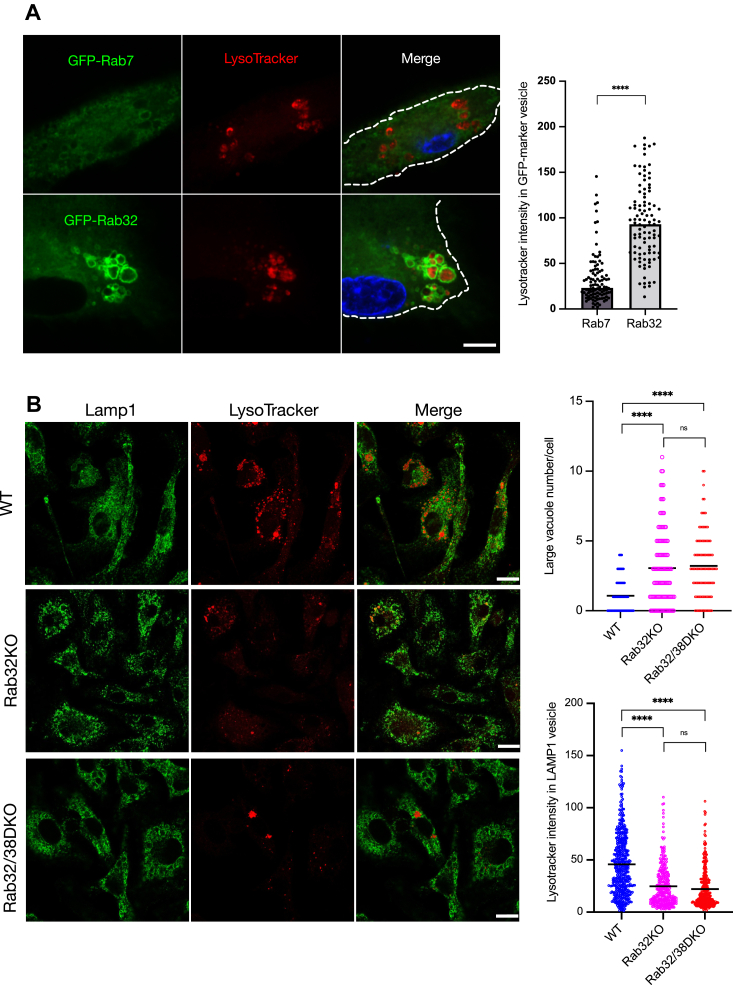
**Rab32 and Rab38 affect the establishment of lysosomes, lysosome-like organelles, or both**. *A*, GFP-Rab7 or GFP-Rab32 expressing macrophages were treated with 50 nM LysoTracker for 1 h. Cells were fixed and observed by confocal fluorescence microscopy. The LysoTracker fluorescence intensity of GFP-Rab7- and GFP-Rab32-positive vesicles was detected using the ImageJ program over 100 vesicles in more than four images at each condition. Scale bar: 10 μm. *B*, bone marrow-derived macrophages isolated from either WT or Rab32 KO or DKO mice were treated with 50 nM LysoTracker for 1 h and fixed with 4% PFA. Cells were labeled with rat anti-Lamp1 antibodies and observed by confocal microscopy. Scale bar: 10 μm. The graph shows the numbers of large vacuoles per cell from over 100 cells in three images in each condition. Large vacuoles were defined by a diameter exceeding 1.5 μm. The fluorescence intensity of LysoTracker in each Lamp1-positive vesicle was collected for over 300 vesicles in three images under each condition tested. These are representative patterns among multiple experiments. Statistical significance was determined by unpaired two-tailed *t* test (∗∗∗∗*p* < 0.0001). DKO, double knockout; Lamp1, lysosomal associated membrane protein 1.

We used LysoTracker, which labels acidic organelles, to observe GFP-Rab32 expressing macrophages (Fig. 7*A*): the inside of the Rab32-positive organelle was labeled, indicating that it is an acidic organelle.

Next, we investigated whether Rab32 and Rab38 affect the organelle’s biogenesis. Macrophages were collected from wildtype and Rab32 and Rab38 double knockout (DKO) or Rab32 single knockout mice. Immunofluorescence using an anti-Lamp1 antibody revealed that the number of Lamp1-positive vacuoles are significantly increased in Rab32 and Rab38 DKO or Rab32 KO macrophages relative to WT macrophages (Fig. 7*B*). These cells were treated with LysoTracker. We found that LysoTracker signals were much lower in Lamp1-positive organelles in DKO or Rab32 KO macrophages compared to WT (Fig. 7*B*). These results indicate that Rab32 and Rab38, especially Rab32, affect the biogenesis of these organelles in macrophage.

**Figure 8:**
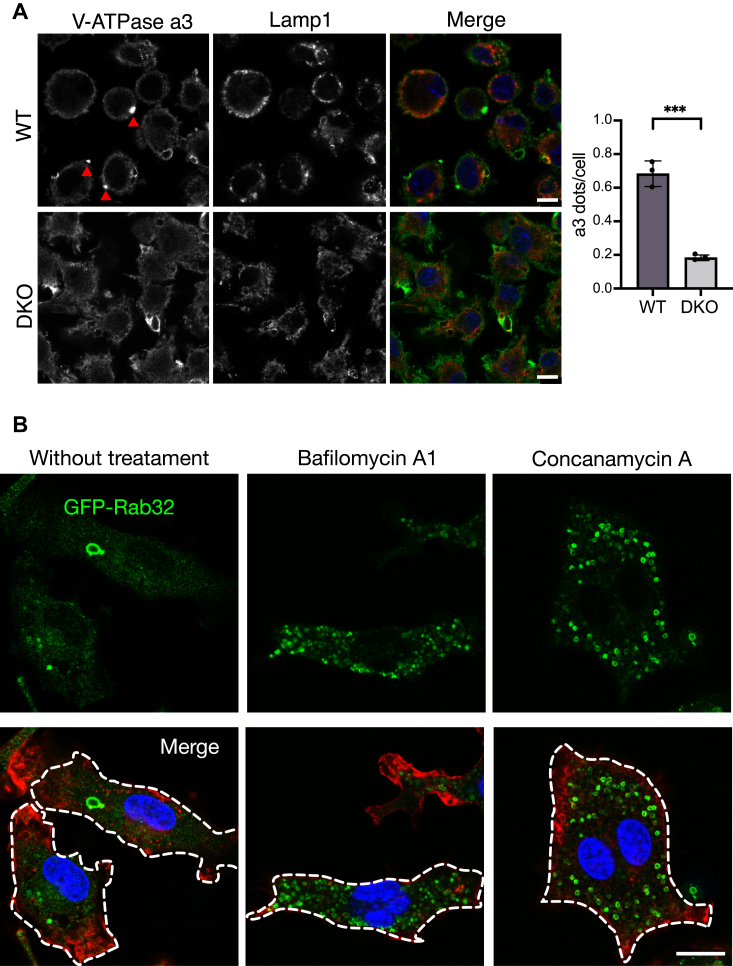
**Lysosomes positive for V-ATPase *a*3 subunit were fewer in number and *a*3 accumulation in plasma membrane were absent in Rab32 and Rab38 DKO osteoclasts**. *A*, BMDMs isolated from either WT or DKO mice were cultured for 3 days and fixed with 4% PFA. Fixed cells were labeled with rat anti-Lamp1, chicken anti-*a*3 antibodies, and DAPI. In the merged image, *green* indicates the *a*3 subunits (Alexa-488), *red* indicates Lamp1 (Alexa-568), and *blue* indicates DAPI. *White arrows* indicate *a*3 signal on the plasma membrane. Red arrows indicate intracellular *a*3 positive structure. Scale bar: 7.5 μm. The number of a3 dots per cell was counted over 50 cells from three images in the graph. ∗∗∗ indicates *p* < 0.001 by unpaired two-tailed *t* test. *B*, macrophages were genetically transfected with GFP-Rab32 and incubated with either 50 nM bafilomycin A1 or 20 nM concanamycin A for 24 h. After fixation, the cells were labeled with actin and observed by confocal fluorescence microscopy. Scale bar: 25 μm. BMDM, bone marrow-derived macrophage; DKO, double knockout; Lamp1, lysosomal associated membrane protein 1.

V-ATPase is a multisubunit proton pump responsible for lysosomal acidification (30). There are four isoforms of the membrane-intrinsic a-subunit, namely *a*1, *a*2, *a*3, and *a*4. Isoform *a*3 is abundantly expressed in osteoclasts and accumulates within lysosome-related organelles. The V-ATPase with isoform *a*3 is relocalized to the plasma membrane of resorption lacuna in fully mature osteoclasts, thereby providing an acidic milieu to facilitate bone resorption. In the macrophages, the V-ATPase with isoform *a*3 is mainly localized to lysosomes in the perinuclear region, and they are recruited to newly formed phagosomes to acidify them. Thus, the V-ATPase with isoform *a*3 plays important roles in both macrophages and osteoclasts (31, 32). We determined that after RANKL treatment of macrophages, some *a*3 colocalized with Lamp1 signals (Fig. 8*A*). We observed mostly single intense punctuated *a*3-positive structures in WT macrophages (Fig. 8*A*, triangle), although this structure’s identity is unknown. Such intense structures were, however, not observed from macrophages derived from Rab32 and Rab38 DKO mice (Fig. 8*A*). These results indicate that Rab32 and Rab38 are involved in functional localization of V-ATPase.

When macrophages were treated with specific inhibitors of V-ATPase, namely bafilomycin A1 or concanamycin A, the number of GFP-Rab32-positive organelles increased significantly (Fig. 8*B*). This observation suggests that proper V-ATPase function contributes to the biogenesis of Rab32-positive organelles.

## Discussion

In this study, we described unique organelles in macrophages that contain Rab32 and organelles that contain both Rab32 and Rab38 in osteoclasts. In macrophages, Rab32 colocalized with the late endosomal and lysosomal proteins, Lamp1 and Lamp2, to characteristic ring-like structures. However, another late endosomal and lysosomal protein, Rab7, was scarcely associated with Rab32-positive organelles. Phagocytosis of several pathogens, including *Salmonella* species bacteria*, Mycobacterium tuberculosis*, and *Legionella* species bacteria reportedly occurred within Rab32-positive phagosomes (33, 34, 35). A portion of phagocytosed fluorescent beads ended up inside Rab32-positive organelles, suggesting that Rab32-positive organelles participate in the endocytic pathway. However, after a longer period, the percentage of beads inside Rab32-positive organelles decreased to around 20% in sharp contrast to Lamp1-positive lysosomes, which contained most of the beads. These results suggest that Rab32-positive organelles can be placed at an intermediate route that leads eventually to lysosomes. Therefore, Rab32-positive organelles in macrophages are likely to be LROs. LROs are organelles that possess certain lysosomal properties, such as an acidic interior and an association with lysosomal proteins but differ from lysosomes in their cell type-specific functions. LROs include melanosomes in melanocytes, dense granules in platelets, and lamellar bodies in alveolar epithelial cells (36). Rab32 and Rab38 are involved in melanosome maturation and regulate melanin synthase transport in melanocytes (37). Hence, chocolate mice express a Rab38 point mutant along with Rab32, and Rab38 DKO mice show hypopigmentation (24, 38, 39, 40) (Tokuda *et al*., submitted).

Our finding that Rab32 and Rab38 colocalize in osteoclasts suggests that they function together in this cell type. This idea is consistent with a previous report that Rab32 and Rab38 have overlapping functions in melanocytes (29). Among all the *Rab* genes, *Rab38* transcript levels showed the highest degree of upregulation during osteoclast differentiation. However, *Rab38* expression was only modestly upregulated on day 3 after RANKL stimulation when *Ctsk* (cathepsin K) is fully upregulated. Rab38 expression reaches its peak only on day 5, which suggests that Rab38 is important in later stages of differentiation. In mononuclear cells, which should be in the early stages of differentiation, cathepsin K, cathepsin B, and TRAP, all localized to Rab32- and Rab38-positive organelles, whereas cathepsin K and cathepsin B are not observed in highly differentiated multinuclear osteoclasts. The accumulation of Rab32- and Rab38-positive LROs around absorption pits suggests that Rab32- and Rab38-positive organelles are ultimately transported to the ruffled border in osteoclasts. Cathepsin K and TRAP should ultimately be transported to absorption pits *via* this pathway. Fully differentiated osteoclasts may be less active in Cathepsin K secretion. In osteoclasts, secretory lysosomes and the ruffled border formed by lysosomal transport have been implicated in LROs (41, 42). Rab32/38-positive LROs are partly positive for Rab7 after RANKL stimulation, which may be consistent with a previous report that Rab7 is also crucial for forming absorption pits (13).

The interiors of the Rab32-and Rab38 LRO are acidic. In the absence of Rab32 and Rab38 in DKO mouse-derived macrophages, the number of Lamp1-positive organelles increased. At the same time, the acidification of Lamp1-positive organelles was lost. The V-ATPase *a*3 subunit is abundantly expressed in osteoclasts while modestly expressed in macrophages (32). However, in Rab32 and Rab38 DKO cells, *a*3 expression is severely reduced in both macrophages. V-ATPase activity suppression by bafilomycin treatment also leads to an increase in Lamp1-positive organelles. Similar phenomena have also been observed in osteoclasts (43). Altogether, Rab32-positive LROs formed appropriately depending on Rab32 (and Rab38) and proper acidification mediated by V-ATPase. Without its proper formation, Lamp1-positive organelle biogenesis was apparently also affected, which implies that LRO formation is closely coupled with lysosomes.

A constitutively inactive Rab32 mutant showed only Golgi localization and not LRO localization (data not shown). This suggests that Rab32 moves back and forth between the Golgi and LRO depending on cycles of activation and inactivation, which is also the case in melanocytes (29). Ergo, Rab32/38 may be transported from the Golgi to form LROs and subsequently transported to the ruffled border of osteoclasts. *HPS* is the human gene that, when mutated, causes Hermansky-Pudlak Syndrome, a disease known to cause leukoderma, hemorrhagic tendency, and pulmonary fibrosis due to lysosomal dysfunction. *HPS* encodes the protein BLOC-3, which functions as a GTP exchange factor for Rab32 and Rab38 (44, 45, 46). Therefore, the functions of Rab32 and Rab38 in osteoclasts may also be affected in these patients. In our accompanying paper on osteoclasts, we describe defects in osteoclast function in Rab32 and Rab38 DKO mice that lead to impairment of bone homeostasis (Tokuda *et al*, submitted). Based on these studies, we believe that the physiological significance of these unique LROs will become clear in time.

## Experimental procedures

### Isolation of mouse bone marrow cells and osteoclast culture

Femurs and tibiae were harvested from 8-week-old male C57BL/6J mice (SLC) as well as from the Rab32 and Rab38 DKO and Rab32 SKO mouse line established using a CRISPR-Cas9 system on C57BL/6J mice (Tokuda *et al*. submitted). Both ends of the bone were cut, and bone marrow cells were collected by washing the inside of the bone with α-MEM with L-glutamine and phenol red (Wako) containing 60 μg/ml kanamycin (Wako). The cells were centrifuged at 250*g* for 5 min, the supernatant was removed, and erythrocyte lysis buffer (150 mM NH_4_Cl, 10 mM KHCO_3_, 0.1 mM EDTA, pH 7.4) was used to resuspend the pellet. Bone marrow cells were isolated by centrifugation at 4 °C for 5 min at 250*g*. Cells were cultured at 37 °C under 5% CO_2_ in α-MEM supplemented with 10% fetal bovine serum (Biowest) and 60 μg/ml kanamycin. Two milliliters of medium containing 10% CMG14-12-derived culture supernatant solution were added to 3.5 cm dishes that were later seeded with 1.5 × 10^6^ cells (47). After 3 days of culture, macrophages were collected using 0.02% EDTA and seeded into dishes. Osteoclasts were induced to differentiate for either 5 or 6 days by stimulating macrophages with 2% CMG14-12-derived culture supernatant solution and 400 ng/ml GST-RANKL, which were prepared as described previously (48). The experiment was approved by Animal Experiment Committee of Osaka University, Graduate school of Dentistry.

### Plasmids and lentiviral transduction

The plasmids used are listed in Table S1. For lentivirus recovery, Plat-E was cultured at 37 °C under 5% CO_2_ in D-MEM (High Glucose) with L-glutamine and phenol red (Wako) supplemented with 10% Fetal Bovine Serum (Gibco) and 100 units/ml penicillin and 100 μg/ml streptomycin. We coated 3.5 cm dishes with 1 ml of 0.01% poly-L-lysine (Sigma), allowed them to stand at room temperature for 5 min, washed them with 1 ml of ultrapure water, and air-dried. The cells were subsequently incubated at 37 °C overnight. On the following day, 1 μg plasmid DNA, 1 μg VSVG plasmid DNA, 8 μl polyethylenimine (1 mg/ml) (MW 25,000; Polysciences), and 200 μl Opti-MEM (Gibco) were mixed together and allowed to stand at room temperature for 20 min before being dropped into the dish. After overnight incubation at 37 °C, the medium was removed and 1.5 ml of α-MEM with 60 μg/ml kanamycin and 10% Fetal Bovine Serum (Biowest) was added, and lentiviruses were collected twice, at 48 and 72 h after transduction. To transduce macrophages, 400 μl lentivirus was incubated with 10 μg/ml polybrene (Sigma) at a final concentration for 3 h in a 3.5 cm dish on the second day of culture. To select for appropriate cells, 2 μg/ml puromycin (Sigma) was added after overnight culture. The cells were cultured for 3 more days before their medium was changed, and the cells were cultured for longer until they grew sufficiently.

### DNA microarray analysis and quantitative PCR

QIAzol Lysis Reagent (Qiagen) was used to extract all RNA from cultured cells. RNA purification and DNA microarray analysis were performed in the DNA Chip Development Center for Infectious Diseases, Research Institute for Microbial Diseases, Osaka University. Whole Mouse Genome DNA Microarray 4 × 44K Ver.2.0 (G4846A) (Agilent Technologies) was used to measure mRNA levels, and an Agilent Microarray scanner G2505C was used for plate scanning. 2505C was used for plate scanning, and feature extraction software (Version 10.7.3.1) was used to quantify the spots.

TRIsure (BIOLINE) was used to extract total RNA from cultured cells. Extracted RNA was used as a template for the reverse transcription reaction following the use of an iScript cDNA Synthesis Kit (Bio-Rad) to produce cDNA. For quantitative PCR analysis, cDNA was used as a template and oligonucleotide primers (Sigma) specific for each gene shown in Table S2 were used. PCR reactions were performed using QuantiTect SYBR Green PCR (Qiagen) on a StepOnePlus Real-Time PCR System (Applied Biosystems). Transcript levels were normalized to those of *GAPDH*.

### Western blotting

Macrophages were cultured in 6 cm dishes and harvested to prepare cell extracts. Cells in each dish were washed three times with PBS (137 mM NaCl, 2.7 mM KCl, 10 mM Na_2_HPO_4_, 1.76 mM KH_2_PO_4_, pH 7.4) before 1 ml PBS was added, and cells were collected using a cell lifter (Genetics Japan). The cell suspension was transferred to a 1.5 ml microcentrifuge tube and centrifuged at 900*g* for 3 min at 4 °C. The cells were suspended with Lysis buffer [50 mM Tris-HCl (pH 7.5), 150 mM NaCl, 1 mM DDT, 1% TritonX-100, and Protease inhibitor cocktail (Roche)]. After standing on ice for 20 min, the supernatant was centrifuged at 20,400*g* for 10 min at 4 °C. The supernatant was suspended in 6× SDS sample buffer (300 mM Tris-HCl, pH 6.8, 12% SDS, 30% glycerol, 0.006% bromophenol blue, 0.6 M 2-mercaptoethanol) and boiled for 3 min. For osteoclasts, on days 3 and 5 of RANKL stimulation, cells were cultured in 48-well plates with RANKL stimulation for either 3 or 5 days, and cell extracts were collected. After the cells on the wells were washed once with PBS, 80 μl of 1× SDS sample buffer (50 mM Tris-HCl, pH 6.8, 2% SDS, 5% glycerol, 0.001% bromophenol blue, 0.1 M 2-mercaptoethanol) was added directly to the wells, and a 1‒200 μl 44 Pipette Tip Natural (QSP) was used to collect the cell extracts and transfer them to an adjacent well. Next, the cell extracts in that well were collected.

The same procedure was repeated to collect cell extracts from a total of 16 wells at a time before transferring these to 1.5 ml microtubes and boiling for 5 min. The amount of protein was measured using a Protein Assay Bicinchoninate kit (Nacalai Tesque) and a NanoDrop-1000 (Thermo Fisher Scientific) at a wavelength of 562 nm. Boiled samples were subjected to SDS-PAGE on a 12.5% polyacrylamide gel using a running buffer (25 mM Tris-HCl, pH 8.3, 191 mM glycine, 0.1% SDS) and transferred to PVDF membranes (GE HealthCare) using transfer buffer (25 mM Tris-HCl, pH 8.3, 192 mM glycine, 20% methanol) in transfer device (NA-150: Nihon Eido) (100 V, 130 mA, 1 h). PVDF membranes were treated with PBS-T (3.2 mM Na_2_HPO_4_, 0.5 mM KH_2_PO_4_, 1.3 mM KCl, 135 mM NaCl, 0.05% Tween 20, pH 7.4) containing 5% skim milk (Morinaga) for 15 min at room temperature. The primary antibody was incubated in a diluted solution of Can Get Signal (TOYOBO) Solution I for 1 h at room temperature, followed by washing with PBS-T for 15 min. Next, the secondary antibody was incubated in a diluted solution of Can Get Signal Solution II for 1 h at room temperature, followed by washing with PBS-T for 15 min. After washing, we performed ECL detection using the Select Western Blotting Detection Reagent (GE HealthCare), and bands of interest were detected using Gene Gnome 5 (Syngene). The band intensity was measured using ImageJ. Antibodies were used at the following dilutions: rabbit anti-Rab32 (27), 1/1000; rabbit anti-Rab38 (27), 1/1000; mouse anti-Rab32 (Santa Cruz, B-4, sc-3901784) 1/200; mouse anti-cathepsin K (Abcam), 1/1000; mouse anti-β-actin (Sigma), 1/10,000; HRP-conjugated anti-rabbit IgG (CST), 1/2000; HRP-conjugated anti-mouse IgG (Southern Biotech), 1/10,000.

### Immunolabeling and microscopy

Cells were cultured in 24-well plates with a 13 mm cover glass. When culturing on dentin sections, cells were cultured in 48-well plates with Dentin Slice, from Ivory, Thin Type (Wako). The fluorescent probe LysoTracker Red DND-99 (Thermo Fisher Scientific) was added 1 h before fixation to a final concentration of 50 nM. Additionally, Magic Red CatB (Immunochemistry Technology, LLC), 1/520, and Magic Red CatK (Immunochemistry Technology, LLC), 1/520, were added 30 min prior to fixation. Magic Red are specific enzyme substrates that fluoresce after cleavage by active cathepsin B or cathepsin K. To inhibit organelle acidification, either 50 nM bafilomycin A1 (TORONTO RESEARCH CHEMICALS) or 20 nM concanamycin A (Sigma) was added to the medium 24 h prior to fixation. Cells were washed three times with PBS, permeabilized with 0.3% TritonX-100/PBS for 2 min at room temperature, washed three times with blocking buffer (1% BSA, 0.1% TritonX-100/PBS), and left in blocking buffer for 1 h at room temperature. The cells were reacted with primary antibody diluted in blocking buffer overnight at 4 °C. After washing three times with 0.02% TritonX-100/PBS, cells were incubated with secondary antibody diluted in blocking buffer for 1 h at room temperature and washed three times with 0.02% TritonX-100/PBS. For Fig. 1*A*, cells were subjected to mild permeabilization with 50 μg/ml digitonin for 10 min, followed by blocking with 0.2% gelatin/PBS. This buffer was also used for antibody dilution. TritonX-100 was neither used in washing nor blocking buffers.

To label actin, the cells were incubated with Acti-stain 555 phalloidin (Cytoskeleton, Inc) diluted 1/625 in 0.2% gelatin/PBS for 30 min at room temperature. Next, the cells were washed three times with 0.02% TritonX-100/PBS, and the cover glass used to seal glass slides using Slow Fade (Invitrogen) with 1 μg/ml DAPI (Sigma).

To label the V-ATPase *a*3 subunit, 0.4% saponin (S0019–25G, Tokyo Chemical Industry Co) was used for permeabilization and 5% normal donkey serum (017–000–121, Jackson ImmunoResearch) was used for blocking. Prepared samples were incubated with primary antibody (chicken anti-*a*3 (49)) diluted 1/500 in blocking buffer [0.4% saponin (S0019–25G, Tokyo Chemical Industry Co), 5% normal donkey serum, and 1% Fetal Bovine Serum (F7524, Sigma-Aldrich)] in PBS either at room temperature for 1 h or 4 °C overnight. After washing three times with blocking buffer, it was incubated with secondary antibody [Alexa Fluor 488-conjugated AffiniPure donkey anti-chicken IgY (IgG) (H + L)] (Jackson ImmunoResearch) diluted 1/500 in blocking buffer and 1 μg/ml DAPI (Sigma) for nuclear fluorescence labeling in blocking buffer with for 1 h at room temperature.

To observe dentin sections, a 200 μm thick Heat Shrinking PLA-plate (TAMIYA) with 8 mm diameter holes was glued to the glass slide. Dentin sections were placed inside the PLA-plate, and a drop of Slow Fade (Invitrogen) with DAPI (Sigma) was added. The top was sealed with a cover glass for observation with a TSC SP8 confocal laser microscope (Leica). The objective lens was an HC PL APO CS2 63 × 1.40 oil immersion lens (Leica). The fluorescent intensity on the line was measured using the ImageJ program. The dilution for each antibody is shown below. rat anti-Lamp1 (Santa Cruz: 1D4B), 1/200; rat anti-Lamp2 rat (DSHB), 1/100; rabbit anti-Rab32 (27), 1/250; rabbit anti-Rab38 rabbit (27), 1/250; mouse anti-Rab32 (Santa Cruz, B-4, sc-3901784), 1/100; mouse anti-Rab38 (Santa Cruz, A-8, sc-3901768), 1/100; rabbit anti-EEA-1 (Cell Signaling), 1/50; mouse anti-cathepsin K mouse (Abcam), 1/50; mouse anti-TRAP (Santa Cruz), 1/50; Alexa Fluor 488-conjugated anti-rabbit (Thermo Fisher Scientific), Alexa Fluor 568-conjugated anti-rabbit (Thermo Fisher Scientific), Alexa Fluor 568-conjugated anti mouse (Thermo Fisher Scientific), and Alexa Fluor 594-conjugated anti-rat (Thermo Fisher Scientific), 1/5000.

### Endocytosis analysis

Cells were cultured in 24-well plates with a 13 mm cover glass. These were cultured with 1.0 μm blue fluorescent FluoSpheres Sulfate microspheres (365/415) (Thermo Fisher Scientific) diluted 1/5000 directly in the culture medium. After fixation at each time point, the cover glass was sealed on the glass slide using Slow Fade. The slides were observed with a TSC SP8 confocal laser microscope (Leica).

## Data availability

The data of this study are available from the corresponding author, T. N., upon reasonable request.

## Supporting information

This article contains supporting information (50, 51, 52, 53).

## Conflict of interest

The authors declare no conflict of interest with the contents of this article.
