# Supplementary figures and images for: Characterization of Rab32- and Rab38-positive lysosome-related organelles in osteoclasts and macrophages
Source: J Biol Chem. 2023 Aug 23;299(10):105191. doi: 10.1016/j.jbc.2023.105191 (PMC10518718; doi:10.1016/j.jbc.2023.105191)

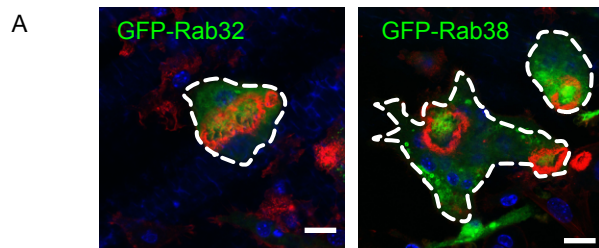

B

Change ratio of expression  
after differentiation

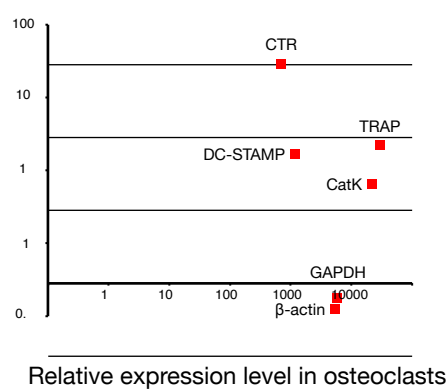

C

|       | Day 3 | Day 6 |
|-------|-------|-------|
| Rab32 | 0.422 | 0.532 |
| Rab38 | 4.87  | 48.1  |

Supplement: Supplemental figure 1 [file mmc1.pdf]

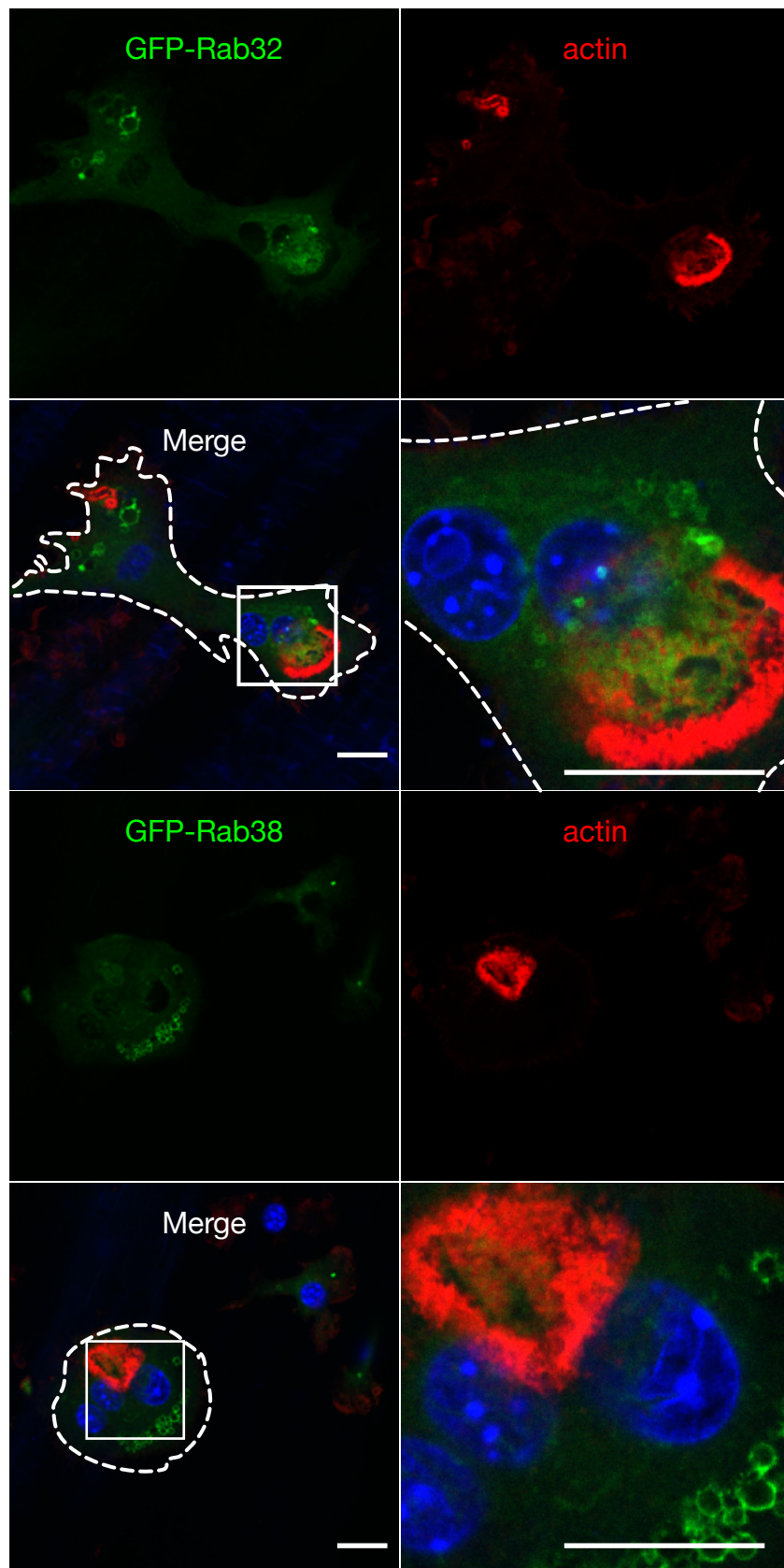

Supplemental figure 2

Supplement: Supplemental figure 2 [file mmc2.pdf]

A

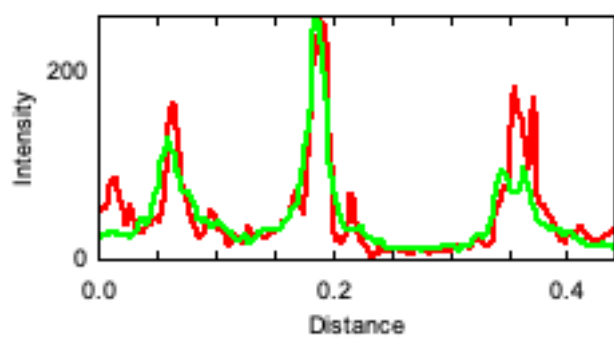

B

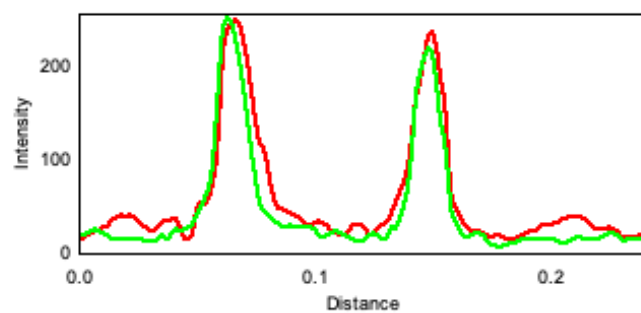

C

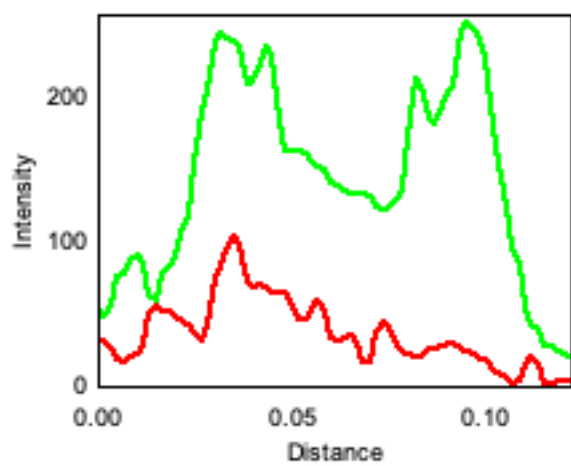

D

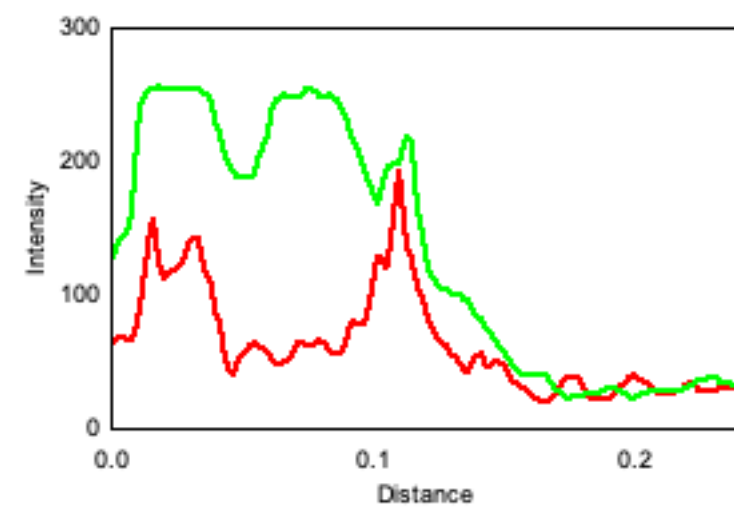

E

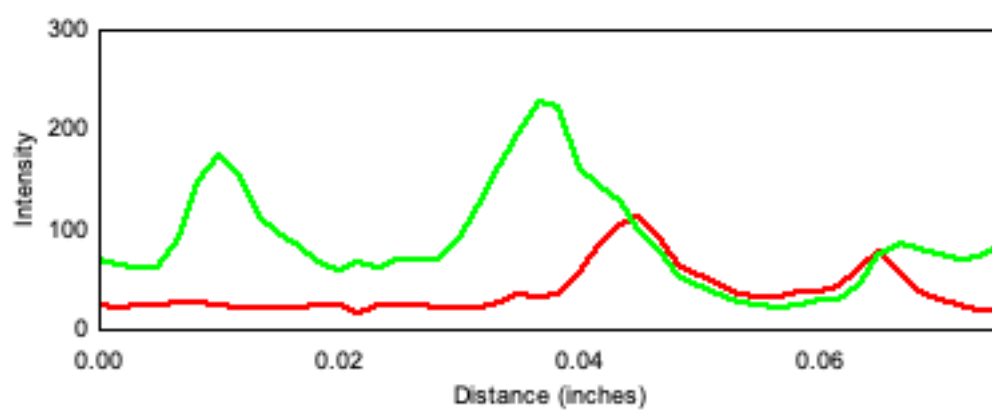

Supplement: Supplemental figure 3 [file mmc3.pdf]

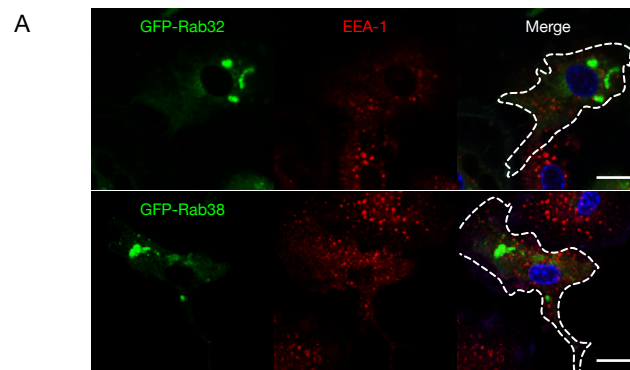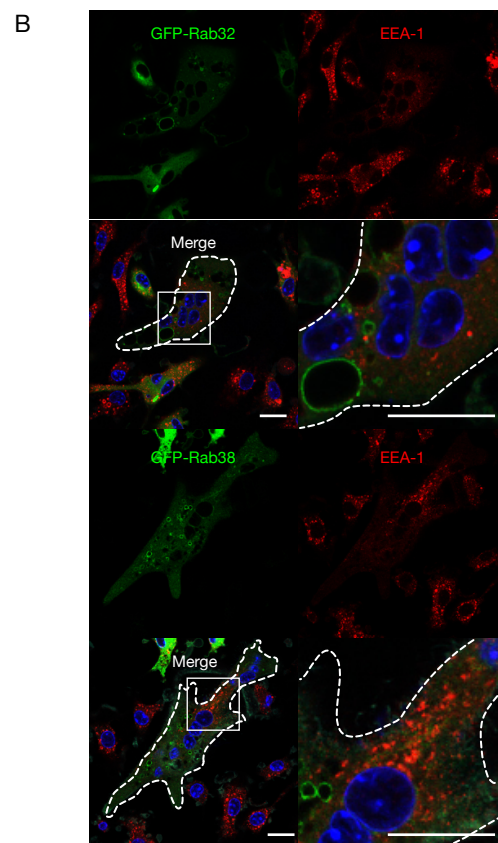

Supplemental figure 4

Supplement: Supplemental figure 4 [file mmc4.pdf]

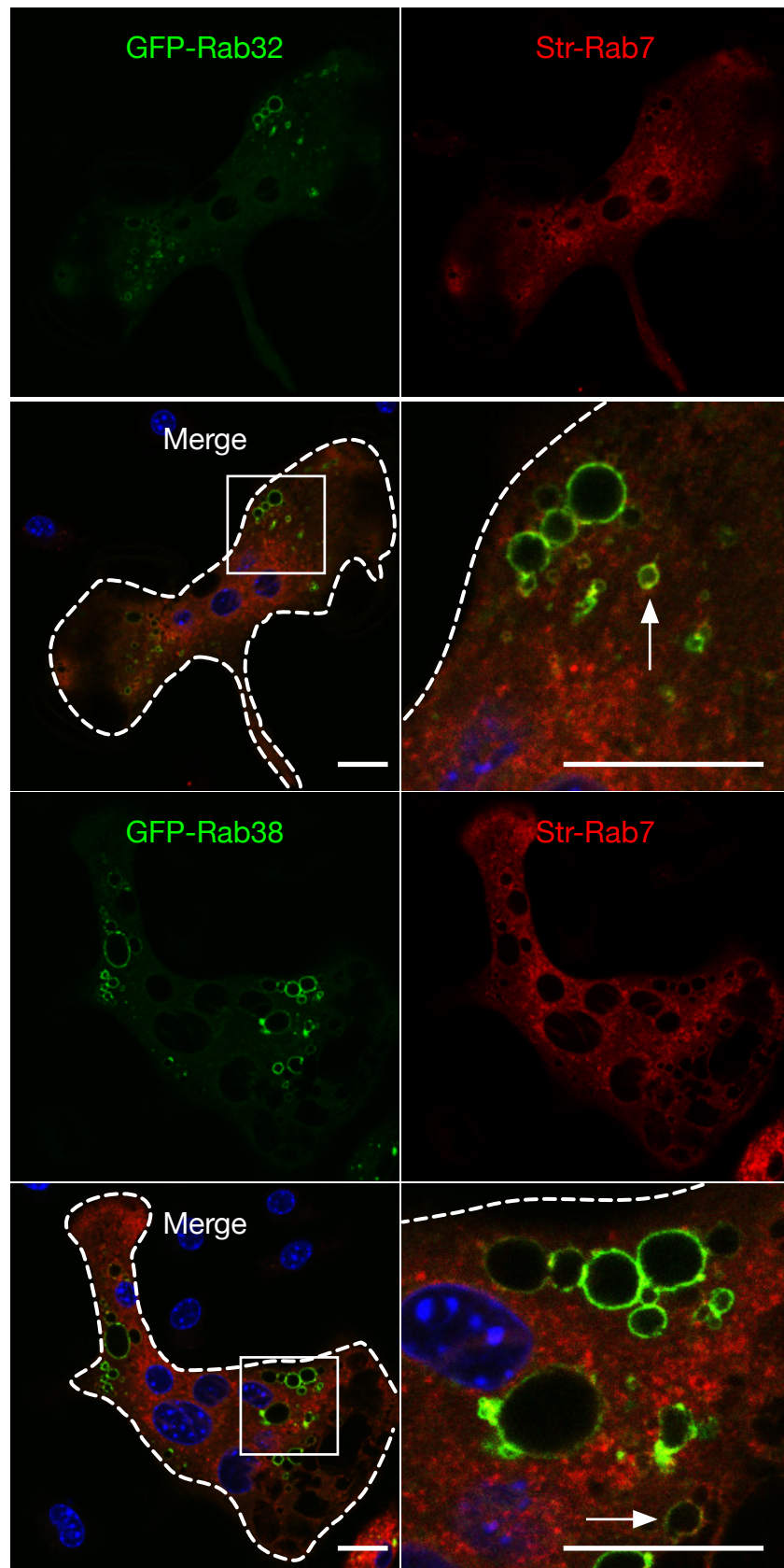

Supplemental figure 5

Supplement: Supplemental figure 5 [file mmc5.pdf]

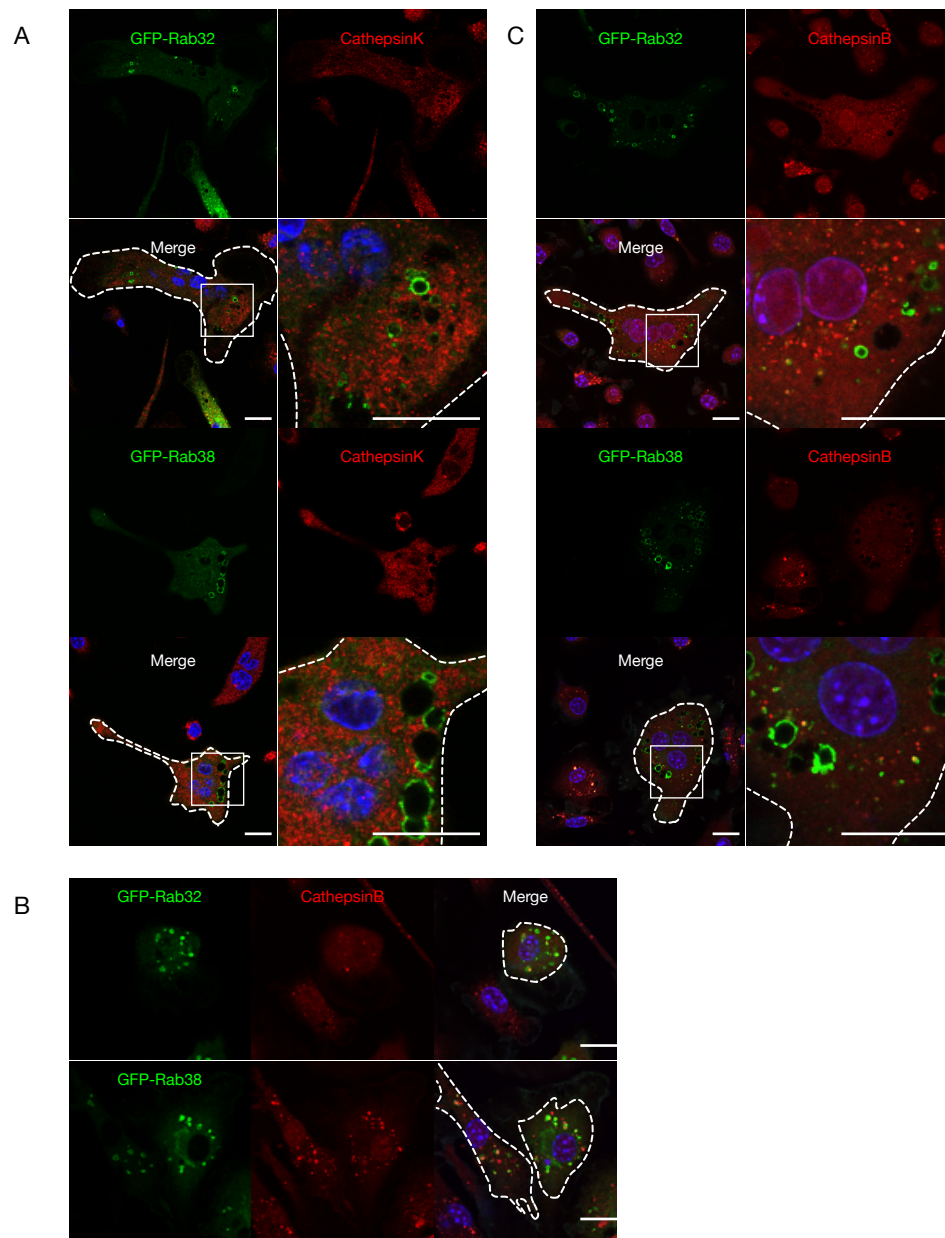

Supplemental figure 6

Supplement: Supplemental figure 6 [file mmc6.pdf]
