# Supplementary material for: Characterization of Rab32- and Rab38-positive lysosome-related organelles in osteoclasts and macrophages
Source: J Biol Chem. 2023 Aug 23;299(10):105191. doi: 10.1016/j.jbc.2023.105191 (PMC10518718; doi:10.1016/j.jbc.2023.105191)
Supplement: Supplemental Tables [file mmc7.pdf]

Supplemental Table 1. Plasmids used in this study.

Plasmid	Reference	Plasmid	Reference
pMRX-puro-EGFP	(50)	pMRX-puro-EGFP-Rab24	(51)
pMRX-puro-EGFP-Rab1A	(51)	pMRX-puro-EGFP-Rab25	(51)
pMRX-puro-EGFP-Rab1B	(52)	pMRX-puro-EGFP-Rab26	(51)
pMRX-puro-EGFP-Rab2A	(51)	pMRX-puro-EGFP-Rab27A	(51)
pMRX-puro-EGFP-Rab2B	(52)	pMRX-puro-EGFP-Rab27B	(52)
pMRX-puro-EGFP-Rab3A	(51)	pMRX-puro-EGFP-Rab28	(51)
pMRX-puro-EGFP-Rab3B	(52)	pMRX-puro-EGFP-Rab29	(51)
pMRX-puro-EGFP-Rab3C	this study	pMRX-puro-EGFP-Rab30	(51)
pMRX-puro-EGFP-Rab3D	(52)	pMRX-puro-EGFP-Rab31/22B	(52)
pMRX-puro-EGFP-Rab4A	(51)	pMRX-puro-EGFP-Rab32	(51)
pMRX-puro-EGFP-Rab4B	(52)	pMRX-puro-EGFP-Rab33A	(51)
pMRX-puro-EGFP-Rab5A	(51)	pMRX-puro-EGFP-Rab33B	(52)
pMRX-puro-EGFP-Rab5B	(52)	pMRX-puro-EGFP-Rab34	(51)
pMRX-puro-EGFP-Rab5C	(52)	pMRX-puro-EGFP-Rab35	(51)
pMRX-puro-EGFP-Rab6A	(51)	pMRX-puro-EGFP-Rab36	(51)
pMRX-puro-EGFP-Rab6B	(52)	pMRX-puro-EGFP-Rab37	(51)
pMRX-puro-EGFP-Rab7	(51)	pMRX-puro-EGFP-Rab38	(51)
pMRX-puro-EGFP-Rab8A	(51)	pMRX-puro-EGFP-Rab39A	(51)
pMRX-puro-EGFP-Rab8B	(52)	pMRX-puro-EGFP-Rab39B	(52)
pMRX-puro-EGFP-Rab9A	(51)	pMRX-puro-EGFP-Rab40A	(51)
pMRX-puro-EGFP-Rab9B	(52)	pMRX-puro-EGFP-Rab40B	(52)
pMRX-puro-EGFP-Rab10	(51)	pMRX-puro-EGFP-Rab40C	(52)
pMRX-puro-EGFP-Rab11A	(51)	pMRX-puro-EGFP-Rab41/43	(51)
pMRX-puro-EGFP-Rab11B	(52)	pMRX-puro-EGFP-Rab42/7B	(51)
pMRX-puro-EGFP-Rab12	(52)	pMRX-puro-EGFP-Rab32QL	this study
pMRX-puro-EGFP-Rab13	(51)	pMRX-puro-EGFP-Rab32TN	this study
pMRX-puro-EGFP-Rab14	(52)	pMRX-puro-Lamp1-mRFP	(53)
pMRX-puro-EGFP-Rab15	(51)	pMRX-bsr-mStr- Rab5A	this study
pMRX-puro-EGFP-Rab17	(51)	pMRX-puro-mStr-Rab7	this study
pMRX-puro-EGFP-Rab18	(51)		
pMRX-puro-EGFP-Rab19	(51)		
pMRX-puro-EGFP-Rab20	(51)		
pMRX-puro-EGFP-Rab21	(51)		
pMRX-puro-EGFP-Rab22A	(51)		
pMRX-puro-EGFP-Rab22B	(52)		
pMRX-puro-EGFP-Rab23	(51)		

Supplemental Table 2. Primers for used for quantitative PCR.

Gene	Primer
GAPDH	5'-AAATGGTGAAGGTCGGTGTG-3'<br>5'-TGAAGGGGTCGTTGATGG-3'
CTSK	5'-CAGCTTCCCCAAGATGTGAT-3'<br>5'-AGCACCAACGAGAGGAGAAA-3'
CTR	5'-CGCATCCGCTTGAATGTG-3'<br>5'-TCTGTCTTTCCCCAGGAAATGA-3'
Rab32	5'-TCGCCCTCAAAGTTCTCAAC-3'<br>5'-ATGTTGCCAAACCGTTCCT-3'
Rab38	5'-CCAAAACTTCTCCTCGCACT-3'<br>5'-TCATGTTTCCAAATCTTTCTTGAC-3'
